# Automated measurement of pineal gland calcification volumes and sleep quality in adults living in costal Ecuador

**DOI:** 10.1055/s-0045-1814399

**Published:** 2026-01-25

**Authors:** Oscar H. Del Brutto, Robertino M. Mera, Emilio E. Arias, Denisse A. Rumbea, Vishal Patel, Pablo R. Castillo

**Affiliations:** 1 Universidad Espíritu Santo, Escuela de Medicina, Samborondón, Ecuador.; 2 Universidad Espíritu Santo, Centro de Investigaciones, Samborondón, Ecuador.; 3 Freenome, Inc., Biostatistics/Epidemiology, South San Francisco CA, United States.; 4 Mayo Clinic College of Medicine, Department of Radiology, Jacksonville FL, United States.; 5 Mayo Clinic College of Medicine, Sleep Disorders Center, Jacksonville FL, United States.

**Keywords:** Pineal Gland, Sleep Quality, Epidemiology

## Abstract

**Background:**

Studies on the association between pineal gland calcification (PGC) and non-breathing sleep-related symptoms are inconclusive.

**Objective:**

The present study aims to evaluate this association in middle-aged and older adults living in rural villages located in coastal Ecuador.

**Methods:**

Community-dwellers aged ≥ 40 years enrolled in the Three Villages Study cohort were interviewed with the Pittsburgh Sleep Quality Index (PSQI) to assess sleep quality and received head computed tomography for automated measurement of PGC volumes. Generalized linear and logistic regression models were used to assess the association between PGC volumes (exposure) and the PSQI score and sleep quality (as separate dependent variables), after adjusting for age and sex.

**Results:**

The study included 1,009 participants (mean age: 56.5 ± 12.6 years; 57% women). The mean volume of PGC was 51 ± 53.5 µL. The mean score of the PSQI was 5.3 ± 2.8 points, with 399 (40%) participants having poor sleep quality. Locally-Weighted Scatterplot Smoothing showed a linear relationship between continuous PGC volumes and PSQI scores. An unadjusted generalized linear regression model showed a significant association between PGC volumes stratified in tertiles and the continuous PSQI score. However, this association lost statistical significance after adjustment for age and sex. The association between tertiles of PGC and poor sleep quality was non-significant in both unadjusted and multivariate logistic regression models.

**Conclusion:**

Study results did not find an association between increased PGC and sleep quality after adjusting for demographics, suggesting that PGC may not necessarily indicate pineal dysfunction but could reflect adaptive physiological mechanisms.

## INTRODUCTION

The main function of the pineal gland is to secrete melatonin, a hormone associated with the regulation of the sleep-wake cycle. 1 Therefore, it seems reasonable to hypothesize that there is a correlation between pineal gland dysfunction and non-breathing sleep-related symptoms. The pineal gland becomes increasingly calcified with age, particularly in individuals with neurodegenerative disorders. 2 3 4 Since calcified pineal tissue is apparently inactive, the severity of pineal gland calcification (PGC) has been used as a surrogate for pineal gland dysfunction, reduced melatonin secretion, and hence, sleep disturbances.

Despite the reported association between PGC and pineal dysfunction, information on the clinical consequences (i.e., sleep-related symptoms) of these calcifications is limited, and firm conclusions cannot be drawn. 5 6 In a preliminary report, we failed to find a significant association between visually-assessed PGC and sleep-related symptoms in a small sample of older adults living in coastal Ecuador. 7 The present study aims to expand upon previous findings by assessing the association between automated measurements of PGC volumes and sleep quality in a larger sample of community-dwelling middle-aged and older adults living in this region.

## METHODS

### Study population

The current study was carried out in three neighboring rural villages located in coastal Ecuador (Atahualpa, El Tambo, and Prosperidad), where previous research has examined correlates of sleep-related symptoms. 8 9 Inhabitants of these populations share: common race/ethnicity (Amerindian ancestry), low levels of schooling, low socioeconomic status, and dietary habits characterized by a diet rich in oily fish, carbohydrates, and fruits, but poor in other types of meat, dairy products, and highly processed foods). The homogeneity of these populations helps reduce the likelihood of hidden confounders. 10 Additionally, these individuals are exposed to 12 hours of sunlight per day throughout the year, shift work is rare, and nighttime light pollution is minimal, thus minimizing circadian misalignments that may introduce variability in the investigated association.

### Study design

Following a cross-sectional design, community-dwellers aged ≥ 40 years residing in the target villages were identified by means of door-to-door surveys, and those who signed a comprehensive informed consent were enrolled in the Three Villages Study. Eligible candidates were invited to undergo high-resolution head computed tomography (CT), and those who received this exam and were interviewed using the Pittsburgh Sleep Quality Index (PSQI) were included. Women of childbearing age received a pregnancy test before the CT, and those who were pregnant had the exam postponed until after delivery. The study was approved by Hospital Clínica Kennedy's Ethics Committee, Guayaquil (FWA 00030727).

### Computed tomography

Non-enhanced CTs were performed using a Philips Brilliance 64 CT scanner (Philips Medical Systems). Datasets were produced with an in-plane resolution of 0.49 mm and a slice thickness of 1.5 mm. Brain structures were segmented using SynthSeg (open source), 11 a deep learning method capable of processing CT data and demonstrating excellent performance compared to FreeSurfer's (open source) conventional segmentation. 12 Using the SynthSeg, a spherical pineal region of interest (ROI) with a radius of 12 mm was defined, centered 20 mm posterior to the center of mass of the 3 ^rd^ ventricle. An expert neuroradiologist verified that this heuristic ROI included the pineal gland and excluded other calcifications across 50 randomly sampled cases. Voxels within the pineal ROI with attenuation exceeding 50 HU were considered to contain calcified components, and the total volume of such voxels was calculated for each participant.

### Sleep quality assessment

Sleep quality was assessed using the PSQI. 13 As previously reported, this scale has been translated from English to Spanish by bilingual members of our group and has been validated and used with success in studies conducted in the currently investigated population. 8 9 This construct classifies individuals as either “good” or “poor” sleepers. The instrument was administered through face-to-face interviews conducted by trained field personnel. The PSQI evaluates seven components, which include sleep duration, sleep disturbances, sleep latency, daytime dysfunction due to sleepiness, sleep efficiency, overall sleep quality, and the use of sleep medications. The maximum possible total PSQI score is 21 points, with ≥ 6 points indicating poor sleep quality.

### Statistical analysis

Data analyses were carried out using the Stata (StataCorp LLC) software, version 18. In unadjusted analyses, continuous variables were compared using linear models, and categorical variables were analyzed using the Chi-squared or Fisher's exact test, as appropriate. Generalized linear and logistic regression models were used to assess the association between PGC volumes categorized into tertiles (as the exposure) and the PSQI score and sleep quality (as separate outcomes), after adjusting for age and sex.

## RESULTS

The present study included 1,009 (65%) of the 1,543 community-dwellers enrolled in the Three Villages Study. The remaining individuals either declined consent, died, or emigrated between enrollment and invitation, had contraindications for CT, or had imaging degraded by artifacts.

**Figure 1:**
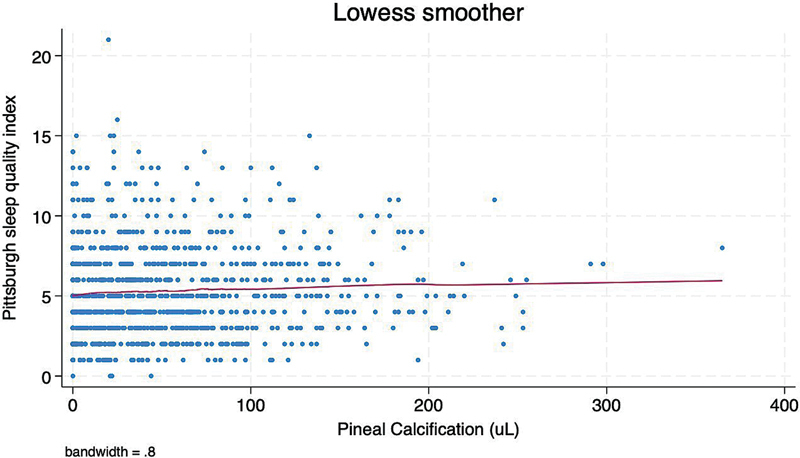
Locally-Weighted Scatterplot Smoothing (LOWESS) graph showing a linear relationship between continuous volumes of pineal gland calcification and scores in the Pittsburgh Sleep Quality Index.

The mean age of participants was 56.5 ± 12.6 (median: 57) years and 573 (57%) were women. The mean volume of PGC was 51 ± 53.5 (median: 35) µL for the entire cohort, with 345 participants allocated to the first tertile (0–18 µL), 328 to the second tertile (19–58 µL), and 336 to the third tertile (59–365 µL). When participants were stratified by the median age, the mean volume of PGC was 45.3 ± 50.5 µL in 506 individuals aged ≤ 57 years, and 56.8 ± 55.8 µL in 503 aged ≥ 58 years ( *p* < 0.001). The mean PSQI score was 5.3 ± 2.8 points (median: 5 points), with 399 (40%) participants scoring ≥ 6 points (indicative of poor sleep quality). Locally-Weighted Scatterplot Smoothing showed a linear relationship between continuous PGC volumes and PSQI scores ( Figure 1 ).

An unadjusted generalized linear regression model showed a significant association between the continuous PSQI score and PGC volumes stratified in tertiles; however, the PSQI score coefficient decreased and lost significance when age and sex were added to the model ( Table 1 ). Age mediated a small portion of the association between the exposure and the outcome. No interaction was detected between age and PGC volumes. The association between PGC volumes stratified in tertiles and poor sleep quality (as a binary variable) was not significant in either an unadjusted logistic regression model or after adjusting for age and sex (data not shown).

**Table 1:** Generalized linear regression models showing associations between volumes of pineal gland calcification (PGC) stratified in tertiles and the continuous Pittsburgh Scale Quality Index (PSQI)

PSQI	β-coefficient	95% confidence interval	*P-value*
PGC 1st tertile	Referent category		
PGC 2nd tertile	0.48	0.05–0.91	0.028*
PGC 3rd tertile	0.43	0.01–0.86	0.050
PGC 1st tertile	Referent category		
PGC 2nd tertile	0.41	−0.20–0.84	0.062
PGC 3rd tertile	0.36	−0.08–0.80	0.110
Age	0.04	0.02–0.05	< 0.001*
Being female	0.25	−0.12–0.61	0.182

Notes: *Statistically significant result. The upper panel shows the unadjusted model, and the lower panel, the model adjusted by age and sex.

## DISCUSSION

This population-based study demonstrates a significant association between PGC and PSQI scores in middle-aged and older adults living in coastal Ecuador. However, this association became non-significant when age and sex were included in the models.

The relationship between PGC and sleep disorders has not been fully explored. In one study, 36 patients with different neurological disorders underwent CT scans and were interviewed with a rather simple questionnaire; the authors found a significant association between PGC severity and sleep disturbances. 5 The same authors measured urine 6-sulfatoxymelatonin (aMT6s) excretion in 26 older adults and found that PGC severity might be used as a surrogate for decreased capacity of the pineal gland to produce melatonin which, in turn, could contribute to sleep-related symptoms. 14 In a subsequent study, 31 patients with primary insomnia underwent polysomnography, urinary aMT6s measurement, and head CTs. Results showed that PGC severity correlated inversely with rapid eye movement (REM) sleep, total sleep time, and sleep efficiency. 6 More recently, solid uncalcified pineal tissue (measured by three-dimensional magnetic resonance imaging) correlated positively with saliva melatonin levels and negatively with sleep quality in 103 healthy individuals. 15 Two additional studies deserve comment. In one of them, urinary aMT6s excretion did not correlate with abnormal polysomnographic patterns in 67 persons, 16 and in another, primary insomnia patients had a smaller pineal volume than controls. 17 Two important pieces of information can be extracted from these studies. First, the degree of PGC correlates with reduced melatonin production. Second, some sleep disturbances might be associated with an impaired ability of the pineal gland to secrete melatonin. However, these studies may have been biased due to small sample size and the recruitment of participants from specialized clinics.

The role of PGC remains an open question. While calcification in other brain structures is typically associated with pathological processes, PGC may represent a physiological adaptation or compensation rather than dysfunction. It has been suggested that PGC could be an incidental finding rather than a marker of neurodegeneration, potentially reflecting normal age-related changes rather than disease progression. 18 In addition, some studies found that significant variations exist in PGC prevalence across populations without corresponding differences in sleep disorders. 2

Another consideration is whether PGC impacts melatonin secretion in a functionally meaningful way. Although studies have linked reduced pineal tissue volume to lower melatonin levels, evidence that PGC itself diminishes melatonin synthesis is limited. 3 4 It is possible that the remaining functional pineal tissue compensates for calcification, maintaining adequate melatonin production despite structural changes.

Furthermore, the findings of our study challenge the assumption that greater PGC necessarily translates to more severe sleep disturbances. The absence of a strong association between PGC and PSQI scores in adjusted models suggests that other physiological or environmental factors play a larger role in determining sleep quality. It is possible that genetic predisposition, lifestyle behaviors, or other neuroendocrine processes exert a stronger influence on sleep regulation than PGC alone.

Our study has limitations. The cross-sectional design precluded the assessment of the direction of the association between the main variables investigated, although biological plausibility suggests that PGC precedes poor sleep quality, since the opposite is unlikely. The current study was carried out in a single geographical region, and our results may not be generalizable to people living at different latitudes. It is possible that some non-explored confounders may have played a role in the observed results. We did not take into account conditions known to favor calcium deposition in the brain (vitamin K deficiency, excessive intake of vitamin D, and genetic disorders). 19 However, these uncommon conditions are unlikely to significantly impact on the findings of the study. In addition, we did not measure urinary aMT6s excretion, which could be a link between PGC and sleep quality. 14 On the contrary, our study has several strengths, including its population-based design, the homogeneity of the study population, the use of a validated instrument for assessing sleep quality, and the automated segmentation of PGC in CT scans by a reliable method. 11 12

Our understanding is that dystrophic calcifications reducing the functional volume of a brain structure would typically result in dysfunction and associated diseases states. However, this does not appear to apply to the pineal gland. Based on the results of the present study, it can be hypothesized that increased PGC volume is not necessarily a marker of pineal gland dysfunction, but rather may reflect mechanisms of compensation or adaptation. Further research, including analysis of sleep chronotypes and polysomnographic assessments, will help elucidate whether PGC correlates with abnormalities of the circadian sleep-wake rhythm.
